# Growing GaN LEDs on amorphous SiC buffer with variable C/Si compositions

**DOI:** 10.1038/srep19757

**Published:** 2016-01-22

**Authors:** Chih-Hsien Cheng, An-Jye Tzou, Jung-Hung Chang, Yu-Chieh Chi, Yung-Hsiang Lin, Min-Hsiung Shih, Chao-Kuei Lee, Chih-I Wu, Hao-Chung Kuo, Chun-Yen Chang, Gong-Ru Lin

**Affiliations:** 1 Graduate Institute of Photonics and Optoelectronics, and Department of Electrical Engineering, National Taiwan University (NTU), No.1, Sec. 4, Roosevelt Road, Taipei 106, Taiwan R.O.C; 2 Department of Electrophysics, National Chiao Tung University, 1001 Ta Hsueh Road, Hsinchu 30010, Taiwan, R.O.C; 3 Department of Photonics and Institute of Electro-Optical Engineering, National Chiao Tung University, 1001 Ta Hsueh Road, Hsinchu 30010, Taiwan; 4 Research Center for Applied Sciences, Academia Sinica, Taipei 115 Taiwan; 5 Department of Photonics and Department of Physics, National Sun Yat-sen University, No. 70, Lien-Hai Rd., Kaohsiung 804, Taiwan; 6 Department of Electronics Engineering, National Chiao Tung University, 1001 Ta Hsueh Road, Hsinchu 30010, Taiwan

## Abstract

The epitaxy of high-power gallium nitride (GaN) light-emitting diode (LED) on amorphous silicon carbide (a-Si_x_C_1−x_) buffer is demonstrated. The a-Si_x_C_1−x_ buffers with different nonstoichiometric C/Si composition ratios are synthesized on SiO_2_/Si substrate by using a low-temperature plasma enhanced chemical vapor deposition. The GaN LEDs on different Si_x_C_1−x_ buffers exhibit different EL and C-V characteristics because of the extended strain induced interfacial defects. The EL power decays when increasing the Si content of Si_x_C_1−x_ buffer. The C-rich Si_x_C_1−x_ favors the GaN epitaxy and enables the strain relaxation to suppress the probability of Auger recombination. When the Si_x_C_1−x_ buffer changes from Si-rich to C-rich condition, the EL peak wavelengh shifts from 446 nm to 450 nm. Moreover, the uniform distribution contour of EL intensity spreads between the anode and the cathode because the traping density of the interfacial defect gradually reduces. In comparison with the GaN LED grown on Si-rich Si_x_C_1−x_ buffer, the device deposited on C-rich Si_x_C_1−x_ buffer shows a lower turn-on voltage, a higher output power, an external quantum efficiency, and an efficiency droop of 2.48 V, 106 mW, 42.3%, and 7%, respectively.

Gallium nitride (GaN) is the most intriguing material beucase it exhibits large bandgap energy and high electronic saturation speed to utilize versatile optoelectronic applications including light-emitting diodes (LEDs)^1^, the laser diodes^2^, solar cells^3^, and field effect transistors^4^. Specially, the blue GaN LED has been investigated to favor its important applications in the flat panel display^5^, the white lighting, and the visible light communication^6^. In order to further enhance the performance of GaN LEDs, the substrate selection has emerged as a new research topic in recent years. The GaN LED has been considered to fabricate on versatile substrates such as the sapphire^7^,^8^,^9^, silicon (Si)^10^,^11^,^12^, zinc oxide^13^,^14^,^15^, silicon carbide (SiC)^16^,^17^,^18^, and GaN^19^,^20^,^21^. In common, the GaN LED is grown on a sapphire substrate because the sapphire substrate shows the high chemical stability at low cost. In 1993, Nakamura *et al.* fabricated the high-power violet InGaN/GaN LED with its output power and external quantum efficiency of 90 μW and 0.15%, respectively^7^. In addition, Hwang *et al.* also utilzied the nonpolar GaN LED grown on the r-plane sapphire substrate^8^. However, the large lattice mismatch between sapphire and GaN is 16% to degrade the device performance.

Alternatively, Yoshida *et al.* employed the AlN film as buffer layer to improve the performance of strain relaxation^9^. The Si substrate becomes another candidate to further decrease the manufacturing cost and large-area fabrication. When the Si substrate is used as the substrate, the GaN LED can be compatible and integrated with Si substrate to form all Si-based optoelectronic devices. In 1998, Guha *et al.* fabricated the ultraviolet and violet GaN LEDs with the peak wavelengths of 360 nm and 420 nm, respectively^10^. Moreover, Tran *et al.* also tried to fabricate the multiple-quantum-well InGaN/GaN LED on Si substrate^11^. In 2006, Dadgar *et al.* successfully grew the crack-free GaN LED on Si substrate by using the AIN buffer with thickness of 5.4 μm. Unfortunately, the lattice mismatch between GaN and Si is larger than that between GaN and sapphire to limit the direct growth of the high-quality and defect-free GaN on Si substrate without additional buffer. Moreover, the large thermal expansion coefficient between GaN and Si also contributes to the defect generation in the LED structure. In order to solve the lattice mismatch between GaN and aforementioned substrates, ZnO is selected because of its superiorities of small lattice mismatch and large-area fabrication. The ZnO was used as n- or p-type layer to combine with the GaN for growing blue LEDs. Hwang *et al.* employed the p-ZnO to grow the GaN LED with a peak wavelength *of* 409 nm^13^. Alivov *et al.* preliminarily employed the n-ZnO/p-GaN to fabricate the LED on 6H-SiC^14^. Rogers *et al.* also utilized the n-ZnO to achieve the hybrid GaN LED with its peak wavelength of 375 nm^15^.

Note that the SiC is another candidate of the buffers to replace Si, ZnO, and sapphire. The SiC materials possess the high thermal stability (4.2 W/cm-K) and good chemical inertness, which provides higher electrical breakdown field than Si to operate at the higher voltages and lower leakage currents. Most importantly, the lattice constant of the SiC substrate is closer to that of the GaN material as compared to that of Si substrate. However, previous studies mainly focused on fabricating the GaN LED on the bulk crystalline SiC such as the 4H- or 6H-SiC^16^,^17^. Scholotter *et al.* reported the growth of blue GaN LED on 6H-SiC bulk substrate with the peak wavelength at 430 nm^16^. Edmond’s group fabricated the GaN LED on 4H- and 6H-SiC bulk substrates with external quantum efficiency as high as 47%^17^. Few reports have mentioned the possibility of the deposited SiC film as a buffer for high-quality GaN epitaxy^18^. In recent years, the crystalline SiC film has been used as the buffer in order to deposit the GaN film^18^,^22^,^23^,^24^,^25^. Takeuchi and co-workers employed the 3C-SiC film as an intermediate layer to grow the GaN LED upon the Si substrate^18^. Yang’s group deposited the crystalline SiC film on Si on insulator substrate for the growth of GaN epilayers^22^. Boo *et al.* reported that the cubic SiC buffer layer grown on (111)-oriented Si wafer by using the chemical vapor deposition (CVD) can be employed to deposit the hexagonal GaN thin films^23^. Wang and co-workers studied the cubic GaN film grown on (001)- and (111)-oriented Si coated with thin flat SiC^24^,^25^. From abovementioned reports, it is concluded that most studies emphasized on the GaN LED grown on crystalline SiC buffer. In general, the crystalline SiC substrate needs the high temperature growth which makes the whole fabrication procedure not only complicated but also expensive for large-area fabrication. In contrast, the a-Si_x_C_1−x_ layer can be deposited on any kinds of substrates at relatively low temperate, which also favors to decrease the lattice mismatch between the GaN film and the Si or sapphire substrate. The a-Si_x_C_1−x_ film can be easily synthesized on large-area substrates with precise tuning on the growth recipe. The fabrication parameter of the a-Si_x_C_1−x_ buffer layer needs not be emphasized so as to effectively reduce the cost of fabrication.

More recently, the flexible and transparent LEDs have been considered for extending their functionality in the future. Figure 1 illustrates that the transparent and flexible LED can be attached on any host substrates, such as flexible metal, glasses, plastic plates, mirrors, etc. However, only organic LED was utilized for this purpose and few reports were emphasized on GaN LED lifted-off from the host substrates. Therefore, the a-Si_x_C_1−x_ buffer is a suitable candidate to develop the transferable and flexible GaN LED for versatile substrates. Nevertheless, there were few works using amorphous Si_x_C_1−x_ (a-Si_x_C_1−x_) buffer for epitaxy of GaN LEDs, and our proposed method can further be applied for the easy transfer of the GaN LED from Si to other substrates. The a-Si_x_C_1−x_ layer has never been used as a buffer for the epitaxy of the GaN LED. In this work, the GaN LEDs grown on a-Si_x_C_1−x_ buffers with different C/Si composition ratios are characterized. In addition, one method to achieve the transfer of the flexible GaN LED on a-Si_x_C_1−x_ buffer is demonstrated in this work. The effects on the strain, quantum efficiency, power, and droop characteristics for the GaN LED grown on SiC/SiO_2_/Si substrate are compared when the Si_x_C_1−x_ film with different C/Si composition ratios is used as the buffers for the GaN LED growth. We further emphasize on the successful epitaxy of the GaN LED deposited on the a-SiC buffer without performance degradation, and develop the procedure for transferring the flexible GaN LED on a-Si_x_C_1−x_ buffer from the SiO_2_/Si substrate to a copper plate for future industrial application. To confirm, we demonstrate the lift-off method to exfoliate the GaN LED on a-Si_x_C_1−x_ buffer and transfer it from the SiO_2_/Si substrate to the copper plate without using laser cutting. The experimental results show that the performance of the GaN LED is not degraded before and after immersing in the buffer oxide etching solution for separating the GaN LED with a-Si_x_C_1−x_ buffer from SiO_2_/Si wafer.

## Results

### Structural and Compositional Analyses of the Nonstoichiometric Si_x_C_1−x_ Buffers

**Figure 2:**
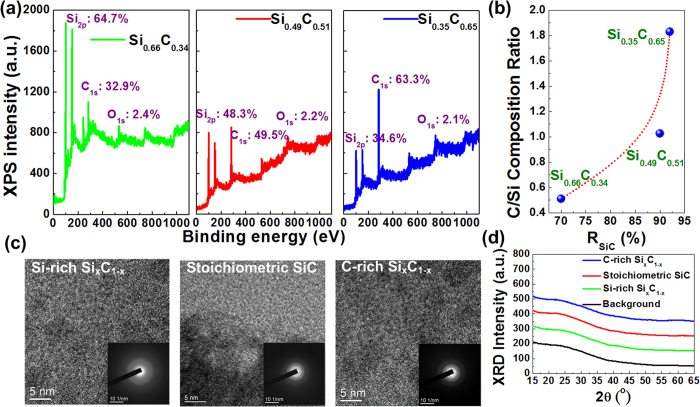
Material characteristic of Si_x_C_1−x_ film. (**a**) The XPS spectra of the Si-rich, the stoichiometric, and C-rich Si_x_C_1−x_ films. (**b**) The C/Si composition ratio of the Si_x_C_1−x_ film as a function of R_SiC_. (**c**) FETEM image, SAD pattern and (**d**) XRD spectra of the Si-rich, the stoichiometric, and C-rich Si_x_C_1−x_ films.

Figure 2(a) shows the C/Si composition ratio of the nonstoichiometric Si_x_C_1−x_ films as a function of the [CH_4_]/[CH_4_+SiH_4_] fluence ratio by the X-ray photoelectron spectroscopy (XPS) analysis. When the [CH_4_]/[CH_4_+SiH_4_] fluence ratio is enlarged from 70% to 92%, the C/Si composition ratio of the nonstoichiometric Si_x_C_1−x_ increases from 0.51 to 1.83. It corresponds to a composition transferring from Si-rich to C-rich condition, as shown in Fig. 2(b). The decomposed energy is inevitably decreased to supply each molecule at constant RF plasma power and higher molecular density. The molecules obtain less energy to make the decomposition rates of SiH_4_ and CH_4_ molecules become gradually indistinguishable with each other. This phenomenon can contribute to the reduction of SiH_4_ decomposition rates. Therefore, the nonstoichiometric Si_x_C_1−x_ film grown with high [CH_4_]/[CH_4_+SiH_4_] fluence ratios reveals the high C/Si composition ratio. Moreover, the composition of the nonstoichiometric Si_x_C_1−x_ can be detuned from Si-rich to C-rich condition by varying the [CH_4_]/[CH_4_+SiH_4_] fluence ratios.

With high-resolution field emission transmission electron microscopy (FETEM) analysis, neither Si nor C clusters can be found in all nonstochiometric Si_x_C_1−x_ samples. The structural phase of these Si_x_C_1−x_ samples are almost amorphous, as shown in Fig. 2(c). However, some blurred diffraction rings can be observed from the selected area diffraction (SAD) analysis of selected area in the stoichiometric and C-rich Si_x_C_1−x_. The diffraction rings in the SAD pattern almost fade out for Si-rich Si_x_C_1−x_ when its structural phase structure is completely amorphous. By contrast, the SAD patterns for stoichiometric and C-rich Si_x_C_1−x_ samples exhibit at least two diffraction rings as a supporting evidence of nano-scale SiC clusters. With the detailed analysis on the diffraction ring, the two diffraction rings for stoichiometric SiC are 0.307 nm and 0.226 nm, which are contributed by (110)-oriented 3C-SiC (0.311 nm) and (200)-oriented 3C-SiC (0.227 nm) phases, respectively. It indicates that the stoichiometric SiC possesses a 3C-SiC like bonding structure. In contrast, the C-rich Si_x_C_1−x_ film reveals d-spacings of 0.373 and 0.222 nm, which are attributed to the (004)-oriented 6H-SiC and the (102)-oriented 4H-SiC, respectively. It also represents that the both 6H- and 4H-SiC based nano-scale phase structures may co-exist in the C-rich Si_x_C_1−x_. Figure 2(d) shows the X-ray diffractometer (XRD) spectra of the Si_x_C_1−x_ films grown at different [CH_4_]/[CH_4_+SiH_4_] fluence ratios to observe their amorphous phase structure. In comparison with the glass background, there is no XRD signal to present the crystallinity for all nonstoichiometric Si_x_C_1−x_ films. This indicates that the phase structures of all nonstoichiometric Si_x_C_1−x_ samples are amorphous from macroscopic observation. However, the nano-scale grains may be still existed in the nonstoichiometric Si_x_C_1−x_ when the selective-area structural analysis observation is performed by using SAD and TEM.

### Structural and Device Performance of the GaN LED grown upon Si_x_C_1−x_ buffer

**Figure 3:**
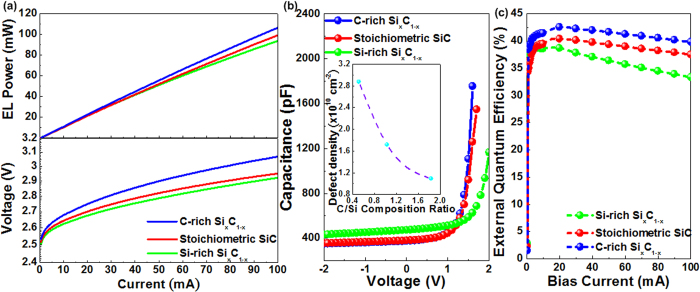
The electrical characteristics of GaN LEDs grown with different C/Si composition ratio based Si_x_C_1−x_ films. (**a**) The V-I (Lower) and P-I (Upper) characteristics, (**b**) C-V characteristics, defect density (Inset), and (**c**) EQE of the GaN LEDs grown with different C/Si composition ratio based Si_x_C_1−x_ films.

The voltage-current (V-I) curves of the GaN LED deposited on the Si_x_C_1−x_/SiO_2_/Si substrate by varying the C/Si composition ratios of the buffered Si_x_C_1−x_ film are shown in the lower part of Fig. 3(a). When the C/Si composition ratio of the Si_x_C_1−x_ buffer increases from 0.51 to 1.83, the turn-on voltage of GaN LED slightly decreases from 2.61 V to 2.48 V. It indicates that the C-rich Si_x_C_1−x_ buffered layer can effectively release the strain of GaN layers. The lattice constants of the 4H- and 6H-SiC (a=0.3073 nm) move closer to that of the GaN (a=0.3186 nm) to further release the strain between n-GaN and Si_x_C_1−x_ films. Therefore, the carriers suffer from less scattering process and easily transport in the n-GaN layer to reduce the turn-on voltage. Moreover, the strain relaxation can effectively decrease the resistance from 3.388 Ω to 2.694 Ω when the buffered Si_x_C_1−x_ film is changed from Si-rich to C-rich condition. The upper part of Fig. 3(a) reveals the power-current (P-I) curve of the GaN LEDs grown on Si_x_C_1−x_ buffers with different C/Si composition ratios. The maximal EL power of the GaN LED increases from 93 mW to 106 mW with the corresponding P-I slope increasing from 0.84 mW/A to 1.02 mW/A when the Si-rich Si_x_C_1−x_ film replaces to the C-rich Si_x_C_1−x_. That is also attributed to the contribution of the releasing strain between the n-GaN/C-rich Si_x_C_1−x_ interface. The structure defect related scattering or trapping probability decreases accordingly. Because of the defect reduction in n-GaN layer, more carriers can be effectively injected into LEDs to improve the tunneling probability under the same bias current. It enables the increasing internal quantum efficiency of the GaN LED grown on the Si_x_C_1−x_/SiO_2_/Si substrate.

To characterize the interfacial defect distribution, the capacitance-voltage (C-V) characteristics of GaN LED are measured and shown in Fig. 3(b). The almost unchanged hysteresis during the detuned sweeping of the biased voltage corroborates that nearly zero C-V hysteresis is contributed by defects and doping impurity in depletion region of the GaN LED grown on all kinds of the Si_x_C_1−x_ buffers. The charge storage of LED can be determined by integrating the C-V hysteresis area over the voltage sweeping range with the following formula^26^,


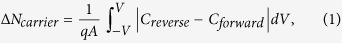


where Δ*N*_*carrier*_ denotes the density of stored carriers in defects of GaN LED with an active area of *A*. The number of carriers stored per defects is defined as the ratio of the integrated area charge density to the estimated area density of defect. As a result, the area defect density is decreased from 2.87 × 10^10^ cm^−2^ to 1.093 × 10^10^ cm^−2^ when the buffered Si_x_C_1−x_ film is changed from Si-rich to C-rich condition in the inset of Fig. 3(b) because the released strain can effectively decrease the defect generation in the GaN LED grown on the C-rich Si_x_C_1−x_ buffer. In general, the defects generated by the threading and edge dislocations co-exist in the GaN film. These two dislocations can be observed from the TEM analysis. In our case, the defects in the GaN LED grown on the a-Si_x_C_1−x_ buffer are mainly contributed by the edge dislocations, which can be confirmed by the high-resolution TEM images. In addition, such high density of defects obtained from C-V analysis is mainly attributed to the quality of GaN MQW LED epitaxial upon the a-Si_x_C_1−x_ buffer. In comparison with the crystalline SiC buffer, there is still observable lattice mismatch as well as strained dislocation occurred during the epitaxy of n-GaN film at very beginning, which inevitably contributes to structural defect generation within grain boundaries so as to scatter or trap the carriers before their radiative recombination.

Consequently, the external quantum efficiency (EQE) of GaN LEDs can be estimated by using the following formula to corroborate with the numerical results given by Z-parameter analysis,


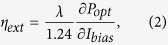


where λ denotes the EL peak wavelength of the GaN LEDs. Figure 3(c) reveals the EQE of the GaN LED grown upon the Si_x_C_1−x_ buffers with different C/Si composition ratios. The maximal EQE of a GaN LED enhances from 38.8% to 42.1% by transferring the Si_x_C_1−x_ buffer from Si-rich to C-rich condition because the strain relaxation between n-GaN and Si_x_C_1−x_ buffer can be effectively improved by detuning the C-rich Si_x_C_1−x_ recipe. Apparently, the decreasing strain at n-GaN/Si_x_C_1−x_ interface also varies the droop of EQE performance. The EQE droop occurs as the recombination mechanism in the GaN LED grown upon Si-rich Si_x_C_1−x_ starts to transfer from radiative to Auger recombination by increasing bias current beyond 10 mA. In particular, the droop of EQE significantly reduces from 15% to 7% when the C/Si composition ratio of Si_x_C_1−x_ buffer increases from 0.51 to 1.83. It indicates that the Auger recombination probability is distinctly suppressed to promote the better radiative recombination mechanism with larger EQE at higher biased condition.

In principle, the EQE droop of GaN LED is also originated from the polarization field, the poor hole transport, and the current spreading. The hole injection in GaN LED is usually hindered as compared to the electron injection because the active region in device is intrinsic or undoped, which increases the poor hole transport and stands alone the material polarization and sheet charge at interfaces^27^. In our case, the GaN LEDs grown on a-Si_x_C_1−x_ buffers with different C/Si composition reveal the same behavior, which exhibit the similar EQE droop induced by the poor hole transportation. This rules out the possibility of hole transportation which dominates the droop variation. In addition, the polarization field in GaN LED is dependent on the growth of GaN film^28^. The stronger polarization field in c-plane GaN LED growth may contribute to the larger EQE droop. In our case, the polarization field in all GaN LEDs remain almost the same as these their n-GaN layers are all grown on a-Si_x_C_1−x_ buffers. Hence the polarization field variation induced EQE droop maybe trivial in the GaN LEDs epitaxial on different a-Si_x_C_1−x_ buffers. On the other hand, the surface roughness induced non-uniform current spreading is also one factor to affect on the low carrier concentration for varying the EQE droop of the GaN LED^29^. From the analysis on EL intensity distribution which represents the uniformity of current spreading, the non-uniformly distributed region of EL intensity shrinks when transferring the buffered Si_x_C_1−x_ film from Si-rich to C-rich condition, where the density of interfacial defect traps gradually reduce to improve the EQE droop. In addition to the Auger recombination, the current spreading uniformity may considerably be another mechanism for the EQE droop variation. Therefore, the lower EQE droop for the GaN LED grown a-SiC buffer is mainly contributed by both the weaker Auger recombination and the better current spreading in devices.

To confirm, the dominated recombination mechanism in a GaN LED can be determined by using the Z-parameter analysis^30^. In principle, the net recombination of the LED in steady state can be described as a function of the injected current


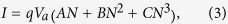


where I and V_a_ denote the injected current and active volume, respectively. In general, the Z-parameter varies between 1 and 3 for different recombination mechanisms. Typically, the Z parameters are 1, 2, and 3 for the defect, radiative, and Auger related recombinations, respectively. In experiment, the Z-parameter can be calculated by plotting the P-I curve of a LED as the derivative of the ln(I) vs. ln(P^1/2^). Goddard *et al.* have defined the Z-parameter using the following formula^30^,


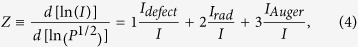


where I_defect_, I_rad_, and I_Auger_ are the defect, radiative, and Auger injected currents, respectively. The EL power is proportional to the current (I_rad_) contributed to the radiative recombination, as described by


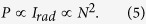


When one of the recombination mechanisms dominates the injected current, the injected current can be approximated by


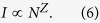


**Figure 4:**
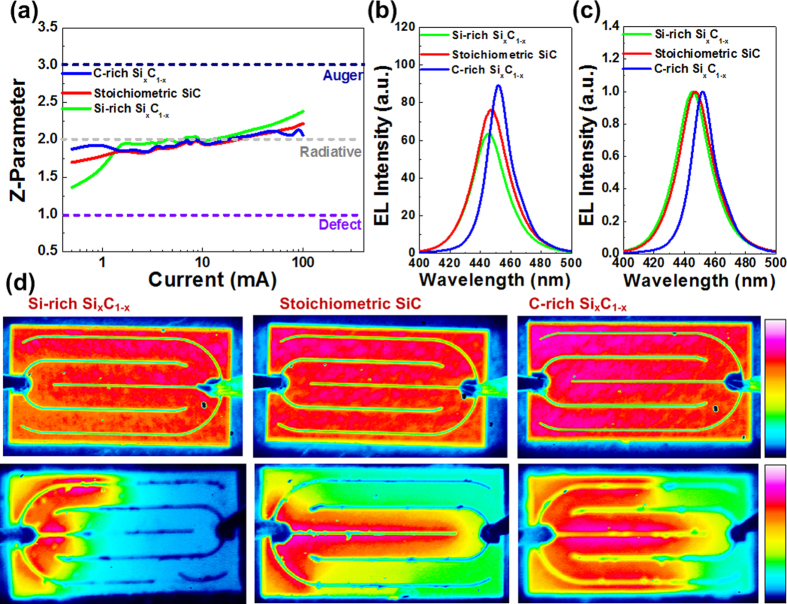
The EL characteristic of GaN LEDs grown with different C/Si composition ratio based Si_x_C_1−x_ films. (**a**) The Z-parameter, (**b**) the EL spectra and (**c**) normalized EL for the GaN LEDs grown with different C/Si composition ratio based Si_x_C_1−x_ films. (**d**) The EL intensity distribution of the GaN LEDs grown with different C/Si composition ratio based Si_x_C_1−x_ films under the operating current of 1 mA (Upper) and 100 mA (Lower).

When the Z-parameter plot can be experimentally obtained from the P-I response of the GaN LED, the Fig. 4(a) shows the Z-parameter curves as a function of biased currents for the GaN LEDs grown upon the Si_x_C_1−x_ with different C/Si composition ratios. At low bias currents, the dominated recombination mechanism in the GaN LED grown on all kinds of Si_x_C_1−x_/SiO_2_/Si substrates is mixed of the defect and radiative recombinations with corresponding Z-parameter ranged between 1 and 2. In all cases, the Z-parameter gradually approaches 2 with increasing biased currents to 1 < I_inj_ < 50 mA (with ±10% deviation). It indicates that the radiative recombination dominates the GaN LED. At the bias current beyond 50 mA, the Auger recombination significantly enhances in the GaN LED grown with Si-rich Si_x_C_1−x_ buffer. Nevertheless, the Auger recombination can still be suppressed in the GaN LED grown upon buffered C-rich Si_x_C_1−x_ film even under the bias current of up to 100 mA. Such the suppression is because of the released strain when it induces the piezoelectric and spontaneous polarization to generate large local spatial field^31^. The field varies the momentums of electrons and holes and contributes to a large spatial separation between the wave functions of carriers, which further decreases the radiative recombination rate and internal quantum efficiency. If the strain can effectively be released in the devices, the generating probability of the Auger recombination can be reduced to further decrease the droop of quantum efficiency.

In general, when the Z-parameter is 2, the radiative recombination dominates all recombination mechanisms in the GaN LEDs. However, Goddard’s group also pointed out that the Z-ptarameter is equivalent to 2 when the co-existed defect and Auger recombinations dominate in GaN LEDs concurrently. In addition, the leakage current and the high level injection are non-ideal effects to make Z-parameter result complicated. If defect and Auger recombinations dominate the Z-parameter at Z = 2 condition, the efficiency of the radiative recombination should be decayed to an extremely low value as both mechanisms are non-rediative processes^32^. Owing to the decreased external quantum efficiency by these two non-radiative phenomena, the luminescent power would be greatly attenuated and associated with a relatively large efficiency droop. This is definitely not in our case, as the measured efficiency droop of the GaN LED on a-Si_x_C_1−x_ buffer is relatively comparable with others reported in previous works in Table 1 33,34,35,36,37,38. For comparison, Ling’s group employed InGaN/GaN MQW as the active layer to deposit on sapphire substrate, which presented the EQE droop of 13% at a biased current of 100 A/cm^2^ 33. Wang *et al.* graded the composition of the InGaN/GaN MQW to improve the EQE droop to only 6% under the operation at 200 mA^34^. More recently, Liu and co-workers replaced the substrate to the Si wafer and obtain the EQE droop of GaN LED of 25% at a bias of 120 mA^35^. Liu’s group added the additional GaN buffer to decrease the lattice mismatch, which further improved the EQE droop of GaN LED to 17% at the bias of 700 mA^36^. To improve the lattice mismatch between the GaN LED and substrate, Hirayama directly used the SiC substrate to decrease the EQE droop of 20% at a bias of 160 mA^37^. Zhao *et al.* simply utilized the GaN substrate to match the lattice constant of GaN film, which contributes to the EQE droop of the GaN LED to 14.3% at a bias of 200 A/cm^2^ 38.

To evaluate the luminescent characteristics of the GaN LED grown on different Si_x_C_1−x_ buffers, the Fig. 4(b) shows the EL spectra of the GaN LED grown upon the Si_x_C_1−x_ films with changing the C/Si composition ratios. Under the bias current of 10 mA, the EL peak wavelengths of the GaN LEDs grown with the different C/Si composition ratios are all located at 450 nm. Because of the band filling effect enhanced by enlarging the bias current^39^, the EL linewidth is slightly enlarged from 19 nm to 26 nm when the Si_x_C_1−x_ buffer is transferred from C-rich to Si-rich condition (which requires higher bias). This mechanism elucidates why the GaN LED grown upon the Si-rich or nearly stoichiometric Si_x_C_1−x_ film easily contributes to a broader spectral linewidth due to higher biased condition. The EL power of the GaN LED decays when the Si content in the Si_x_C_1−x_ buffer increases. The EL spectra of the GaN LED also red-shifts its central wavelength from 446 nm to 450 nm when the stoichiometry of the Si_x_C_1−x_ buffer is changed from Si-rich to C-rich condition, as shown in Fig. 4(c). In general, the blue-shifted wavelength is correlated with both the electric field in InGaN/GaN quantum well and the associated quantum confined Start effect (QCSE) under high biases^40^. In previous work, Xu *et al.* compared the InGaN/InGaN LED with the InGaN/GaN LED and observed the mitigated blue-shift on peak wavelength when the electric field in InGaN/InGaN quantum well reduces^40^. When the Si-rich Si_x_C_1−x_ buffer layer induces higher stain to result in more defects in the InGaN/GaN quantum well, the GaN LED grown upon the Si-rich Si_x_C_1−x_ buffer needs higher bias to overcome the defect trapping for achieving same EL power, which inevitably results in higher internal field in the InGaN/GaN quantum well so as to induce a larger blue-shift on peak wavelength. In principle, the strain relaxation suppresses the piezoelectric components with different polarizations to further decrease the QCSE^41^. As a result, when the Si_x_C_1−x_ buffer is transferred from Si-rich to C-rich condition, the spectral blue-shift almost diminishes as strain and structural defects decrease concurrently.

To characterize the distribution uniformity of the mapped EL intensity as well as bias current^42^, the 2D EL intensity contours of the GaN LED grown upon the Si_x_C_1−x_ buffers with different C/Si composition ratios were measured under the same biased current. It helps to observe if the injected carrier transportation is uniformly distributed between contacts, as shown in Fig. 4(d). When Si-rich Si_x_C_1−x_ film is used, the color of non-uniformly distributed EL intensity contour on the GaN LED surface widely spreads from orange to red, as shown in the upper part of Fig. 4(d). It represents that the carriers are unable to effectively transport along the region ranged between anode and cathode. This phenomenon is mainly attributed to the defect trapping effect as varied by the C-V analysis of the GaN LED. When the defects trap carriers in the lower EL region, the carriers require higher voltage to overcome the potential well around traps. This is because the biased current may contribute to the non-radiative and Auger recombinations. In particular, the Auger recombination will dominate the EL intensity under high biased current. The variation among the EL intensity distributions for all GaN LEDs grown upon different Si_x_C_1−x_ buffers is less distinguishable when the radiative recombination dominates under lower biased currents, as shown in upper part of Fig. 4(d). In contrast, the Auger mechanism arises to degrade the recombination process under highly biased operation. It effectively decreases the EQE to decay the EL intensity. From the lower part of Fig. 4(d), when GaN LEDs grow upon the buffer Si_x_C_1−x_ under the biased current as high as 100 mA, the Auger recombination occurs to differentiate the variation on EL intensity distribution. With Si-rich Si_x_C_1−x_ buffer, the non-uniform distribution of the EL intensity is observed with its contour color broadly spreading from blue to red. Especially, the strongest EL intensity is close to the anode region because the strain induced Auger recombination greatly suppresses the injected carrier transportation within a very short distance. In comparison, the C-rich Si_x_C_1−x_ buffer essentially releases the interfacial strain, decreases the Auger recombination, and improves the radiative recombination. Such a non-uniform distribution of the EL intensity shrinks when the Si_x_C_1−x_ buffer is transferred from Si-rich to C-rich condition, where the trapping density of the interfacial defect gradually reduces to improve both the intensity and uniformity of the EL distribution. In the lower part of Fig. 4(d), the EL intensity distribution can smoothly spread from anode to cathode by improving carrier transportation with suppressed Auger recombination. When the strain is released by growing the GaN LED upon the lattice matched buffer, the EL becomes more efficient to provide higher power with smaller droop, as confirmed from the Z-parameter analysis.

**Figure 5:**
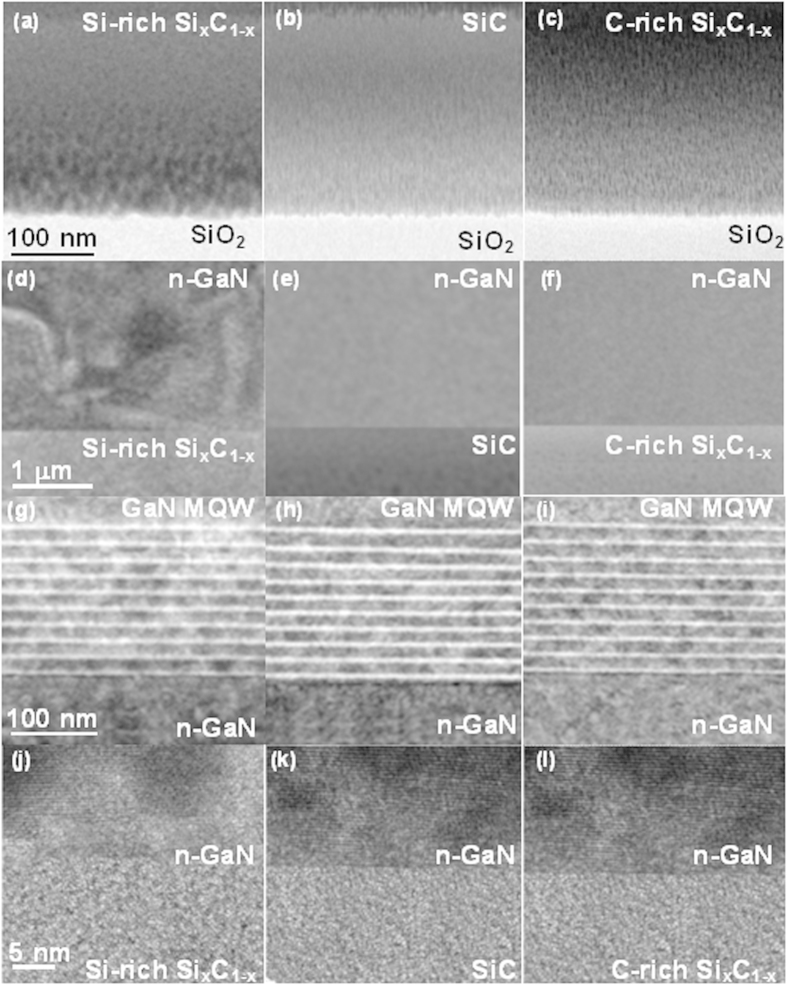
The TEM characteristic of GaN LEDs grown with different C/Si composition ratio based Si_x_C_1−x_ films. 1^st^ Row: TEM images of the interfaces between (**a**) Si-rich, (**b**) stoichiometric, (**c**) C-rich Si_x_C_1−x_ buffers and the SiO_2_/Si substrate. 2^nd^ Row: TEM images of the n-GaN layers grown on (**c**) Si-rich, (**d**) stoichiometric, (**e**) C-rich Si_x_C_1−x_ buffers. 3^rd^ Row: TEM images of the same p-GaN/InGaN-MQW/n-GaN layers epitaxial on (**g**) Si-rich, (**h**) stoichiometric and (**i**) C-rich SiC buffers. 4^th^ Row: High-resolution TEM images of the interface between the n-GaN layers and (**j**) Si-rich, (**k**) stoichiometric, (**l**) C-rich Si_x_C_1−x_ buffers.

Figure 5 shows the TEM images of the a-Si_x_C_1−x_ buffers on SiO_2_/Si substrate (first row), the n-GaN layers on different a-Si_x_C_1−x_ buffers (second row), the InGaN MQW structures on n-GaN/a-Si_x_C_1−x_/SiO_2_/Si (third row) and the high-resolution interfacial images between n-GaN layers and a-Si_x_C_1−x_ buffers (fourth row). From the first row of Fig. 5, it is seen that all a-Si_x_C_1−x_ buffers on SiO_2_ exhibit amorphous structure; however, the Si-rich a-Si_x_C_1−x_ further non-uniformly stacked along the surface normal, indicating its randomly distributed grains at different regions, as shown Fig. 5(a). In contrast, both the stoichiometric SiC (see Fig. 5(b)) and C-rich Si_x_C_1−x_ (see Fig. 5(c)) buffers present relatively fine nano-grains in the whole layer to facilitate the epitaxy of n-GaN. In second row of Fig. 5, the TEM images reveal the structural perfection of n-GaN layers grown upon a-Si_x_C_1−x_ buffers. Figure 5(d) shows that the n-GaN layer exhibits significant grain domains with abundant boundary imperfections owing to the misfit lattice induced dislocation loops. On the contrary, the stoichiometric SiC and C-rich Si_x_C_1−x_ buffers provide more small nano-grains to help the epitaxy of n-GaN with better crystalline quality. Therefore, few structural defects in the n-GaN layer grown on the stoichiometric SiC (see Fig. 5(e)) and C-rich Si_x_C_1−x_ (see Fig. 5(f)) buffers are observed after synthesis. In the third row of Fig. 5, the TEM images of the same p-GaN/InGaN-MQW/n-GaN layers epitaxial on Si-rich, stoichiometric and C-rich SiC buffers are shown. When Si-rich Si_x_C_1−x_ film serves as a buffer, the epitaxial GaN LED shows amorphous structure initially, which inevitably contributes to the structural defect generation such that the performance of the GaN MQW LED is somewhat degraded accordingly, as shown in Fig. 5(g). In comparison, both the epitaxial n-GaN MQW LEDs grown on stoichiometric SiC (see Fig. 5(h)) and C-rich Si_x_C_1−x_ (see Fig. 5(i)) buffers exhibits clearer MQW layer structure with fewer structural defects but some inhomogeneous shadows can still be observed among layers. In more detail, as the a-Si_x_C_1−x_ buffer changes to C-rich condition, the lowest row in Fig. 5(j) shows that the epitaxial n-GaN film upon Si-rich a-Si_x_C_1−x_ remains disordered lattice at beginning, and the distribution uniformity on composition of the n-GaN is the worst among all cases. With the use of stoichiometric SiC buffer, the crystallinity of n-GaN near interface slightly improves so as to effectively reduce the generation of structual defects, as shown in Fig. 5(k). The relatively ordered lattice structure is observed in n-GaN layer shown in Fig. 5(l) by using C-rich a-Si_x_C_1−x_ buffer, which leads to the lowest strain as well as structural defect density so that the dislocation can be minimized in the GaN MQW LED grown upon the n-GaN layer. This is the evidence attributed to the improved V_turn-on_, EL, EQE, and droop property of the GaN LED on C-rich Si_x_C_1−x_ buffer with the best strain relaxation.

**Figure 6:**
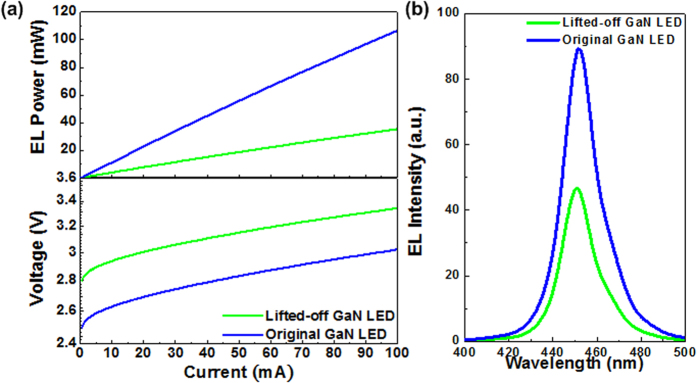
The EL characteristic of the lifted-off GaN LEDs. (**a**) The V-I (lower) and P-I (upper) responses and (**b**) The EL spectrum of the lifted-off GaN LED on a-Si_x_C_1−x_ buffer transferred onto the copper substrate and the original GaN LED on a-Si_x_C_1−x_ buffer.

At last, to further characterize if the performances of the GaN LED is degraded after lift-off process, the I-V and P-I curves of the GaN LED on a-Si_x_C_1−x_ buffer lifted-off on the copper substrate are measured and shown in Fig. 6(a). The lifted-off GaN LED on a-Si_x_C_1−x_ buffer exhibits a turn-on voltage of 2.8 V, an EL power of 35 mW at bias of 100 mA, and the corresponding P-I slope of 0.35 W/A after transferring onto the copper substrate. The Fig. 6(b) shows the EL spectrum obtained at a bias of 10 mA, which reveals the peak wavelength at 446 nm. In particular, the long-term lift-off process in BOE solution could slightly damage the GaN LED to induce more surface states and defects. As compared to the original GaN LED, the lifted-off GaN LED requires higher turn-on voltage but delivers lower optical power. Such an operation also leads to a higher internal electric filed across the MQW structure to induce the stronger quantum confined Stark effect, which effectively contributes to a slightly blue-shifted peak wavelength of EL for the lifted-off GaN LED.

## Discussion

The epitaxy of GaN LED upon the a-Si_x_C_1−x_ buffer grown by low-temperature PECVD on the SiO_2_/Si substrate is demonstrated. When the [CH_4_]/[CH_4_+SiH_4_] fluence ratio is increased from 70% to 92%, the Si_x_C_1−x_ film increases its C/Si composition ratio from 0.51 to 1.83 with the corresponding fractional index (x) reducing from 0.66 to 0.35. The XRD analyzed that the phase structures of all nonstoichiometric Si_x_C_1−x_ samples are amorphous; however, the SAD analysis reveals some blurred diffraction rings from the stoichiometric and C-rich Si_x_C_1−x_. Two SAD rings diameters observed for stoichiometric SiC are 0.307 nm and 0.226 nm, as contributed by (110)- and (200)-oriented 3C-SiC structures, respectively. In contrast, the C-rich Si_x_C_1−x_ film reveals d-spacings of 0.373 and 0.222 nm as attributed to the (004)-oriented 6H-SiC and the (102)-oriented 4H-SiC, respectively. When the Si_x_C_1−x_ buffer changes from Si-rich to C-rich condition, the turn-on voltage of GaN LED slightly decreases from 2.61 V to 2.48 V. It indicates that the C-rich Si_x_C_1−x_ buffer can effectively release the strain of GaN epitaxial layers when the lattice constants of the co-existed 4H- and 6H-SiC in C-rich Si_x_C_1−x_ move closer to that of the GaN. Moreover, the C-V analysis declares that the area density of defects is decreased from 2.87 × 10^10^ cm^−2^ to 1.09 × 10^10^ cm^−2^ when Si-rich SiC buffer replaces to C-rich one when the released strain effectively reduces the defects in the GaN LED grown on lattice matched C-rich Si_x_C_1−x_ buffer. The maximal EL power of the GaN LED increases from 93 mW to 106 mW, and the corresponding P-I slope also increases from 0.84 mW/A to 1.02 mW/A when the Si_x_C_1−x_ buffer is transferred from Si-rich to C-rich condition. The n-GaN/Si_x_C_1−x_ interface defect related scattering or trapping probability decreases accordingly. The Z-parameter analysis shows that the Auger recombination significantly decreases with released stain in GaN LED. Therefore, the maximal EQE of a GaN LED enhances from 38.8% to 42.1%, accompanied with its droop suppressing from 15% to 7% and its EL spectra red-shifted from 446 nm to 450 nm by transferring Si_x_C_1−x_ buffer from Si-rich to C-rich content. When the Si_x_C_1−x_ buffer transforms from Si-rich to C-rich condition, the spectral blue-shift becomes insignificant as the structural defects decrease. When Si-rich Si_x_C_1−x_ film is used, the non-uniform distribution of the EL intensity contour on the GaN LED surface widely spreads from orange to red. It represents that the carriers are unable to effectively transport along the region ranged between anode and cathode. This non-uniform distribution of EL intensity shrinks when the Si_x_C_1−x_ buffer is transferred from Si-rich to C-rich condition, where the trapping density of the interfacial defect gradually reduces to improve both the intensity and uniformity of the EL distribution. This GaN LED grown upon SiC/SiO_2_/Si substrate can be easily lifted-off for applying to the flexible and transparent LED applications.

## Methods

### Fabrication and Measurement of GaN LED on Si_x_C_1−x_/SiO_2_/Si substrate

For the buffered Si_x_C_1−x_ deposition, the 500-nm thick Si_x_C_1−x_ film with different C/Si composition ratios was grown upon the 1-μm thick thermal SiO_2_ coated Si substrate by using a hydrogen-free low-temperature PECVD system. This synthesis was employed with a mixed gaseous recipe of Argon-diluted silane (Ar-diluted SiH_4_) and methane (CH_4_) without hydrogen carriers. The Ar-diluted SiH_4_ fluence was controlled at 150 sccm. The fluence ratio defined as R_SiC_=[CH_4_]/([CH_4_]+[SiH_4_]) was detuned as 70%, 90%, and 92% to fabricate the Si-rich, the stoichiometric, and the C-rich buffer Si_x_C_1−x_, respectively. The working pressure and the RF plasma power were set at 0.3 torr and 100 W, respectively. In addition, the substrate temperature was fixed at 500 °C, which is below the criterion for synthesizing crystalline SiC during deposition. The bonding information and composition ratio of the Si_x_C_1−x_ buffer were analyzed by using the XPS with an Mg K_α_-line radiation at 1256.3 eV. The structural phase of the nonstoichiometric Si_x_C_1−x_ film was characterized by using an XRD (X’Pert PRO, PANalytical). The preferred orientations of nano-grains in nonstoichiometric Si_x_C_1−x_ were determined by using the high-resolution FETEM (JEM-2100, JOEL).

**Figure 7:**
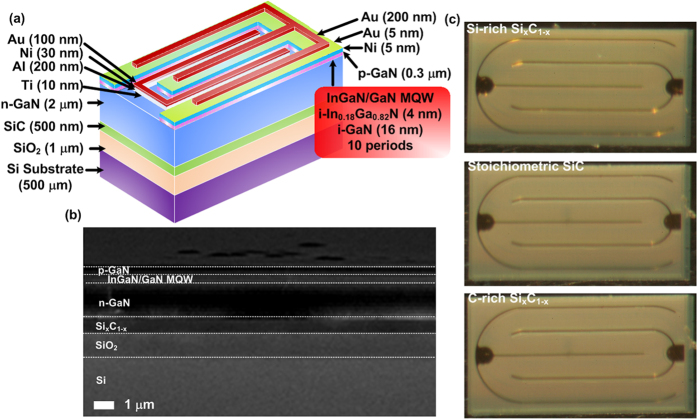
The structure of the GaN LED on SiC/SiO_2_/Si substrates. (**a**) The structure diagram of the GaN LED on SiC/SiO_2_/Si substrates (**b**) The SEM image of the GaN LED devices grown on the SiC/SiO_2_/Si substrate. (**c**) The photograph of the GaN LED devices grown on the SiC/SiO_2_/Si substrate with Si-rich (Upper), the stoichiometric (Middle), and C-rich (Lower) Si_x_C_1−x_ films.

To fabricate the GaN LED on Si_x_C_1−x_/SiO_2_/Si substrate as shown in Fig. 7(a), the GaN LED structure was grown on the Si_x_C_1−x_/SiO_2_/Si substrate by using a MOCVD system. The epitaxial procedure was entrusted to a local GaN LED manufacturer. The GaN LED structure consists of a 2-μm thick n-type GaN bottom layer at first, a 0.2-μm-thick InGaN/GaN multiple quantum well active layer (10 periods with 4-nm thick i-In_0.18_Ga_0.82_N and 16-nm thick i-GaN), and then a 0.3-μm-thick p-type GaN top layer. The transparent Ni/Au (5 nm/5 nm) contact layer was formed by using e-beam evaporation to serve as the ohmic contact on the p-type GaN layer. Subsequently, a 200-nm thick Au contact was patterned on top of the semi-transparent layer to serve as the p-electrode. On the other hand, the Ti/Al/Ni/Au with thickness of 10 nm/200 nm/30 nm/100 nm was successfully deposited onto the exposed n-type GaN layer to serve as the n-type electrode. The cross-sectional SEM analysis of the GaN LED grown upon Si_x_C_1−x_ buffer was performed to scale the thickness and to characterize the uniformity of device structures, respectively. From the SEM image, the thicknesses of the GaN LED, the Si_x_C_1−x_ film, and the SiO_2_ layers are determined as 2.5 μm, 0.5 μm, and 1 μm, respectively, as shown in Fig. 7(b). This SEM image also reveals very clear n- and p-typed LED layered structures with excellent uniformity. It indicates that the GaN LED can successfully deposit on the amorphous Si_x_C_1−x_ covered SiO_2_/Si substrate. Such uniform epitaxy relies strictly on the formation of SiO_2_ layer via the wet-oxidation of the Si substrate, whereas any other kinds of SiO_2_ films formed by gas- or solid-phase deposition cannot meet the demand of broad-area uniformity.

**Figure 8:**
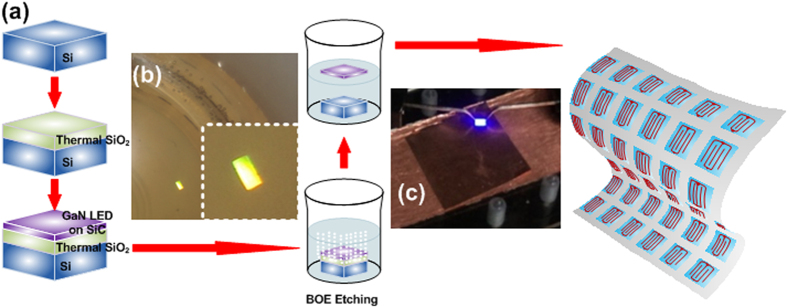
Lift-off process for the GaN LED grown upon a-Si_x_C_1−x_/SiO_2_/Si substrate. (**a**) Fabrication procedure for lifting off the GaN LED. Inset: the two photographs show that (**b**) the GaN LED on a-SiC buffer being lifted off under a buffer oxide etching (BOE) solution, and (**c**) the exfoliated GaN LED on a-SiC buffer transferred on copper substrate.

Figure 7(c) shows the microscopic photograph of the GaN LED grown on the different Si_x_C_1−x_ buffers with changing C/Si composition ratios. The interdigitated electrode structure was designed to enhance the carrier transportation, in which the anode has three fingers and the cathode has two fingers so as to increase the transportation paths. In addition, both the anode and cathode electrodes were allocated on the top surface to avoid the inconvenience caused from the LED package. In order to transfer the GaN LED onto other substrates, the GaN LED on a-Si_x_C_1−x_ buffer needs to be lifted off from a SiO_2_/Si wafer, as shown in Fig. 8. The entire GaN LED/a-Si_x_C_1−x_/SiO_2_/Si sample was immersed in a buffer oxide etching (BOE) solution to remove the SiO_2_ buffer. Afterwards, the GaN LED on a-Si_x_C_1−x_ buffer was clearly separated from the SiO_2_/Si wafers and floated upon the aqueous BOE solution. By gradually diluting the aqueous BOE solution with pure deionized water, other substrates can be used to hold the floated GaN LED on a-Si_x_C_1−x_ buffer subsequently. To distinguish the carrier recombination mechanisms from one another, the I-V curves of GaN LEDs grown upon the buffered Si_x_C_1−x_ with different C/Si composition ratios were measured by programmable electrometer (2400, Keithley). To realize the interfacial defect feature, the capacitance characteristic of the GaN LEDs was measured by a capacitance meter (4280A, HP) with the operating frequency of AC signal at 1 MHz. The EL spectrum ranging from 300 to 850 nm was measured at a biased current of 10 mA, and the EL power was measured using a calibrated power meter with an integral sphere containing a large-area Si detector.

## Additional Information

**How to cite this article**: Cheng, C.-H. *et al.* Growing GaN LEDs on amorphous SiC buffer with variable C/Si compositions. *Sci. Rep.* **6**, 19757; doi: 10.1038/srep19757 (2016).

## Figures and Tables

**Figure 1:**
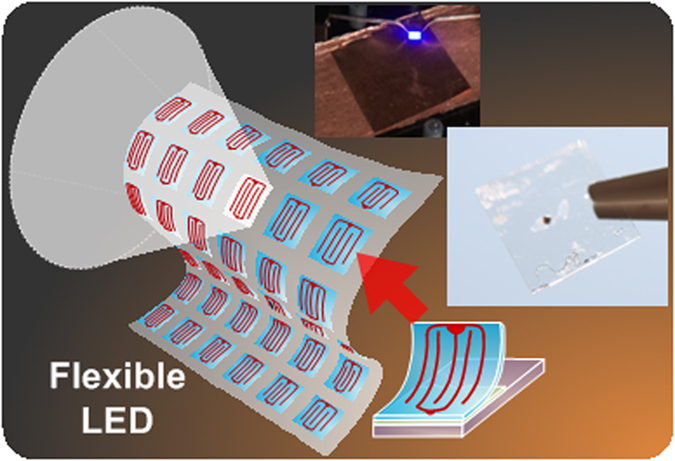
The transparent and flexible LED. The transparent and flexible LED can be attached on any host substrates.

**Table 1:** The performance of GaN LED on versatile substrates in recent years.

Substrate	Structure	EQE Droop	[Ref.]
Sapphire	MQW	13%@ 100 A/cm^2^	[33]
Sapphire	Graded-composition MQW	6% @ 200 mA	[34]
Si	MQW	25%@ 120 mA	[35]
Si	MQW with adding the additional GaN buffer	17.3%@ 700 mA	[36]
SiC	MQW	20%@160 mA	[37]
GaN	MQW	14.3%@200 A/cm^2^	[38]
